# PARP1 and Poly(ADP-ribosyl)ation Signaling during Autophagy in Response to Nutrient Deprivation

**DOI:** 10.1155/2019/2641712

**Published:** 2019-06-09

**Authors:** José Manuel Rodríguez-Vargas, Francisco Javier Oliver-Pozo, Françoise Dantzer

**Affiliations:** ^1^Poly(ADP-ribosyl)ation and Genome Integrity, Laboratoire d'Excellence Medalis, UMR 7242, CNRS/Université de Strasbourg, Institut de Recherche de L'Ecole de Biotechnologie de Strasbourg, 300 bld. S. Brant, CS10413, 67412 Illkirch, France; ^2^Cellular Biology and Immunology, Instituto de Parasitología y Biomedicina López Neyra, CSIC/IPBLN, CIBERONC, Parque Tecnológico de Ciencias de la Salud de Granada, 17 Avenida del Conocimiento, 18016 Armilla, Spain

## Abstract

Autophagy is considered to be the primary degradative pathway that takes place in all eukaryotic cells. Morphologically, the autophagy pathway refers to a process by which cytoplasmic portions are delivered to double-membrane organelles, called autophagosomes, to fuse with lysosomes for bulk degradation. Autophagy, as a prosurvival mechanism, can be stimulated by different types of cellular stress such as nutrient deprivation, hypoxia, ROS, pH, DNA damage, or ER stress, promoting adaptation of the cell to the changing and hostile environment. The functional relevance of autophagy in many diseases such as cancer or neurodegenerative diseases remains controversial, preserving organelle function and detoxification and promoting cell growth, although in other contexts, autophagy could suppress cell expansion. Poly(ADP-ribosyl)ation (PARylation) is a covalent and reversible posttranslational modification (PTM) of proteins mediated by Poly(ADP-ribose) polymerases (PARPs) with well-described functions in DNA repair, replication, genome integrity, cell cycle, and metabolism. Herein, we review the current state of PARP1 activation and PARylation in starvation-induced autophagy.

## 1. Introduction

ADP-ribosylation is a posttranslational modification of proteins with a prominent function in the regulation of diverse biological processes such as, among others, chromatin dynamics, gene transcription, and cell response to DNA damage. Utilizing the oxidized form of NAD^+^ as a substrate, Poly(ADP-ribose) polymerase (PARP) (17 members) catalyzes the covalent attachment of ADP-ribose units onto glutamate, aspartate, tyrosine, lysine, and serine residues of target proteins [1]. Based on their enzymatic activity, PARP enzymes are defined as either mono-ADP-ribose polymerases because they add a single ADP-ribose unit onto their targets (PARP3, vPARP, PARP6, tiPARP, PARP8, PARP10, PARP11 PARP12, PARP14, and PARP15) or Poly(ADP-)ribose polymerases when they create linear or branched Poly(ADP-ribose) chains (PAR chains) (PARP1, PARP2, PARP5a, and PARP5b). No enzymatic activity has been identified for PARP13 while mono-ADP-ribosylation of ubiquitin has been proposed for PARP9 in heterodimerization with the histone H3 ligase Dtx3L (see Table 1) [1, 2].

PARP1, the founding member of the family, has been long defined as a central DNA damage-responsive element required for the maintenance of genome integrity. In response to DNA strand interruptions inflicted by various genotoxic agents, PARP1 promotes an immediate and local production of Poly(ADP-ribose) chains needed for chromatin relaxation through the addition of negatively charged ADP-ribose units onto histones and necessary to coordinate the spatiotemporal assembly of PAR-binding proteins including DNA repair proteins, transcription factors, DNA- and RNA-binding proteins, and intrinsically disordered proteins [3]. As fundamentally important as PAR synthesis in stress response is the following degradation of the ADP-ribose chains by families of ADP-ribose hydrolases. While some enzymes preferentially degrade Poly(ADP-ribose) chains (the macrodomain containing PARG endowed with exo- and endoglycohydrolase activities or ARH3), the removal of the last protein ADP-ribose bond is mediated by a panel of recently identified enzymes including ARH3, TARG1, macroD1, macroD2, NUDT16, or ENPP1 [4].

Owing to its decisive role in DNA repair, the inhibition of PARP1 has emerged as a prominent therapeutic option in cancer treatment, either to potentialize the cytotoxic action of chemotherapy or radiation therapy or to target repair-deficient tumors [5]. To date, three PARP inhibitors have been FDA approved for the treatment of ovarian or breast cancer, namely, Olaparib, Rucaparib, and Niraparib.

The last decades have helped to expand the knowledge on other members of the PARP family and the number of functions of PARP proteins in different cellular pathways. The effects of PARP1 activation (considered the major producer of Poly(ADP-ribose)), its autoPARylation, and PARylation of different targets (which is called transPARylation or heteroPARylation) are translated to cellular responses in several different ways.

Although the majority of functions mediated or regulated by PARPs are related to cellular stress response as DNA damage, it is well known that recent investigations have increased the number and importance of documented PARP-mediated functions and PARylation in noncellular stress situations (see Table 1). The great majority of PAR has a nuclear localization where it will act as a potent posttranslational modification of proteins related to maintenance of genome integrity, gene expression, or cell cycle. However, the cytoplasm has been described, including various organelles, as a large receptor of Poly(ADP-ribose) and single molecules of ADP-ribose. Moreover, PARylated proteins can be transferred to the cytosol, interfering in many different biochemical processes [6]. Noncovalent binding of free Poly(ADP-ribose) is considered a factor of high relevance in the function of the targeted protein. In conclusion, the consequences of PARylation, PAR signaling, or the biochemical interaction of PARP members with partner proteins affect many cellular events ranging from genome homeostasis to vital cellular functions such as cell proliferation, differentiation, metabolism, prosurvival pathways, and programmed cell death.

For some years, it has also become increasingly appreciated that PARP1-catalyzed Poly(ADP-ribosyl)ation regulates cell survival and stress adaptation programs, with among them, the well-conserved self-eating mechanism of cell survival termed autophagy. Herein, we concentrate on the most recent discoveries to encompass the different mechanisms by which PARP1 activity operates in autophagy during periods of nutrient deprivation.

## 2. PARP-Dependent Bioenergetic Changes: NAD^+^ Catabolism and Downstream

PARP1 is considered the main guardian of the genome, able to synthesize up to 85%-90% of the PAR polymer that is needed to maintain cell homeostasis. While the contribution of PARP2 could be considered up to 10%-15%, contribution of other PARPs seems insignificant compared to the total Poly(ADP-ribose) pool. Biochemically, PARP1 constitutes the majority of NAD^+^ catabolic activity in the cells, depleting NAD^+^ to 20% of its normal levels within minutes upon DNA damages [7]. NAD^+^ provides a direct link between the cellular redox status and the control of signaling events, since it is considered an oxidoreductase cofactor in cell physiology and acts as a substrate for a wide range of enzymes. NAD^+^ could be considered to be metabolic fuel in high competence for three kinds of proteins: NAD^+^-dependent protein deacetylases or Sirtuins, Poly(ADP-ribose) polymerases, and transcription factors. Depending on the metabolic context in which the cell is located, the competition for NAD^+^ will be unbalanced towards one pathway or another. Generally, NAD^+^ requirement in the case of PARPs, mainly DNA-dependent PARPs, is higher and is accompanied by a high amount of ATP to complete the synthesis and PARylation of various acceptors. Therefore, PARP overactivity will be triggered in a situation of ATP depletion and promotes a scenario that will compromise the cells. Glycolysis is probably the most immediate energy pathway compromised during PARP overactivation. NAD^+^ is an important cofactor in metabolism; the reduction of NAD^+^ to NADH is essential for the glyceraldehyde 3-phosphate (GAPDH) step of glycolysis and multiple steps in the tricarboxylic acid cycle (TCA). All those situations that could compromise glycolysis immediately impair the major source of ATP production in cells, used, for example, in the cycle to regenerate NAD^+^, and the Krebs cycle. Since the major source to create secondary metabolites and antioxidant precursors is blocked, this creates a negative feedback loop that compromises cell survival [7, 8].

Inside the nucleus, we could focus on the high competence for NAD^+^ in PARP1/SIRT1, cleaving NAD^+^ to produce nicotinamide and ADP-ribosyl products and the enzymes related with Poly(ADP-ribose) recycling such as Poly(ADP-ribose) glycohydrolases (PARGs) and ADP-ribose hydrolase 3 (ARH3). SIRT1 is a NAD^+^-dependent type III histone deacetylase member of the Sirtuin family. Sirtuin proteins catalyze the reaction of NAD^+^ with acyl lysine groups to remove the acyl modification from substrate proteins, resulting in the final production of deacetylated lysine, nicotinamide, and 2′-*O*-acetyl-ADP-ribose (*O*AADPr). This deacetylation integrates cellular NAD^+^ metabolism into a large spectrum of cellular processes, such as cell metabolism, cell survival, cell cycle, apoptosis, DNA repair, and mitochondrial homeostasis [9, 10]. *O*AADPr is considered a second messenger linked with decreased ROS-mediated stress, gene silencing, and ion channel activation [10]. On the other hand, several studies report that PARPs and Poly(ADP-ribosyl)ation can modulate mitochondria from the nucleus through PAR translocation, depletion of NAD^+^ pools, and epigenetic regulation of nuclear genes that are involved in mitochondrial DNA transcription or repair. Short PAR chains and single units of ADP-ribose (ADPr) can be produced from the coordinate actions of PARPs and PARG, which cleavage Poly(ADP-ribose) to free monomers of ADPr. Several data suggest that PARP1-induced loss of ATP requires PARG activity. Under specific scenarios of PARP1 hyperactivation, PARG-dependent production of the single unit of ADPr can exit from the nucleus and interfere with ATP production in mitochondria, promoting nonenzymatic ADP-ribosylation of cell death proteins and metabolic cofactors [4, 6, 7].

Free units of ADPr and *O*AADPr have potential signaling functions in cytoplasm, suggesting mitochondrion-related enzymes as putative targets. The existence of enzymes capable of metabolizing these second messengers suggests that their cellular concentrations may be targeted in metabolism in several pathophysiological situations such as neurodegenerative diseases, acute brain injury, or cancer [4, 6]. Many enzymes target *O*ADDPr and Poly(ADP-ribose) preventing unnecessary effects in the nucleus and cytoplasm. *O*ADDPr is cleaved to ADPr by macroD1, macroD2, and ARH3 enzymes in the nucleus; moreover, in mitochondria, the interconnected activity of PARG/ARH3 degrades short Poy(ADP-ribose) chains to ADPr in the mitochondrial matrix, preventing the heteroPARylation of AIF (apoptosis-inducing factor), mitochondria collapse, and PARthanatos cell death [8].

In the last 30 years, a superfamily of enzymes has gained prominence in the field of Poly(ADP-ribosyl)ation and PAR-mediated biology. These ADPr hydrolases, called NUDIX hydrolases, are considered the major regulators of the intracellular levels of ADPr. The NUDIX superfamily is found in all classes of organisms among eukaryotes, bacteria, and viruses [11]. NUDIX hydrolases are basically pyrophosphohydrolases that act on substrates with the general structure NDP-X (nucleoside diphosphate linked to some moiety, X). Various cellular compounds include the canonical structure NDP-X and also contain oxidized and canonical nucleoside di- and triphosphates, nucleotide sugars, and alcohol, presenting potential toxic properties; so, originally, NUDIX enzymes were proposed to function in housecleaning controlling the availability of intermediates in metabolism [11]. NUDIX hydrolyze ADPr to adenosine monophosphate (AMP) and ribose 5-phosphate in an Mg^2+^-dependent reaction (or a similar cofactor, e.g., Zn^2+^), thereby limiting free ADPr accumulation. In human cells, members of the NUDIX family often exhibit substrate selectivity for specific nucleotide derivatives; NUDT9, an ADPr pyrophosphatase, is highly specific for the mitochondrial ADPr pool, while NADT5, an ADP-sugar pyrophosphatase, only has preference for cytosolic ADPr and other ADP-sugar conjugates; both enzymes catalyze the production of AMP [12, 13]. Recent studies have also demonstrated that NUDIX are potential metabolizing enzymes of the byproduct of Sirtuin activity, *O*ADDPr, producing AMP and *O*-acetylated-ribose-5-phosphate [11]. These studies allowed describing an active crosstalk between mitochondrial PARG/ARH3 Poly(ADP-ribose) metabolisms and subsequently altering the levels of GTP and ATP.

Mitochondrial AMP phosphorylation by adenylate kinase 3 (AK3) and low NAD^+^ levels causes depletion in mitochondrial GTP, compromising mitochondrial fusion. On the other hand, high levels of AMP also increase AMP : ADP ratios, which lead to the activation of AMPk kinases. AMPk phosphorylates the mitochondrial fusion factor or MFF inducing Drp1 translocation from cytosol to mitochondria causing constriction and fission. The final situation results in excessively fragmented mitochondria and eventually leads to mitophagy [14]. Additionally, better understanding of mitochondrial profiles of Poly(ADP-ribosyl)ation and mono(ADP-ribosyl)ation and the complexities of AMPk in relation to mitochondrial dynamics should be taken up in future studies. The crosstalk between nuclear DNA-dependent NAD^+^, energy collapse dependent of PARP1 overactivation, and AMPk activity in response to the proautophagy stimulus will be analyzed in other sections of this review.

PARP1 and SIRT1 compete for a limited nuclear pool of NAD^+^, and of course, each activity could guide the other one, leading to diverse consequences for cells. Different *in vivo* studies have demonstrated the metabolic crosstalk in important models of differentiation, neurogenesis, inflammation, and cancer. PARP inhibitors increase intracellular NAD^+^ levels, promoting specific nuclear deacetylase activity of SIRT1 but not affecting other SIRTs located in cytoplasm or mitochondria; however, the negative correlation was only found under physiological conditions in specific scenarios such as muscle differentiation [15]. In the case of PARP2, new data reflects no evident competence for NAD^+^ with SIRT1 and shRNA-mediated depletion of PARP2 produces little effect on NAD^+^ balance; however, there is a demonstrated physical interaction between both proteins. *Parp2*^−/−^ mice exhibit elevated SIRT1 expression with decreased acetylation of SIRT1 targets FOXO1 and PGC-1*α* which are transcriptional regulators of mitochondrial bioenergetics due to the fact that 63% of mitochondrial localized proteins contain lysine acetylation sites [16]. Indeed, PARP2 deletion in mice produces increased mitochondrial biogenesis in upregulation genes involved in mitochondrial respiration, antioxidant precursor activity, and lipid oxidation such as tpn1, SDH, UCP2, or MCAD [16]. Thus, any consideration of selective PARP2 inhibition in tumor cells and during metabolic unbalance will require better understanding of the exact mechanism of PARP2-dependent metabolic regulation.

DNA-dependent PARPs can be considered transcriptional modulators of several metabolic pathways. PARP1 and PARP2 could be considered important precursors of the activity of different transcription factors that modulate mitochondrial oxidative phosphorylation and lipid oxidation such as PPAR*γ*, FOXO1 by direct PARylation of them, and their cofactors or modulators, consuming high amounts of NAD^+^ and ATP [17]. However, the mechanism whereby PARPs and PARylation transcriptionally mediate metabolic transcription factors remains an unexplored field.

## 3. Cell Death Modulation by PARP1 during Energy Stress

Over the course of the years, different groups have demonstrated that the influence of PARP activity goes beyond the nucleus and directly impacts NAD^+^ metabolism, energy pathways, and oxidative metabolism. Many studies have concluded that in a scenario of energy depletion guided by oxidative stress, PARPs potentiate cell survival or cell death pathways [4, 18]. Reversible PARylation is a pleiotropic regulator of various cellular functions, but uncontrolled PARP activation may also lead to cell death. Moreover, noncovalent PARylation or MARylation (PTM by monomers of ADP-ribose) could be considered as an effective cytosolic posttranslational modification of diverse MAPk kinases and mitochondrial cell death factors, protecting cells from acute DNA damage and stress conditions [18].

**Figure 1:**
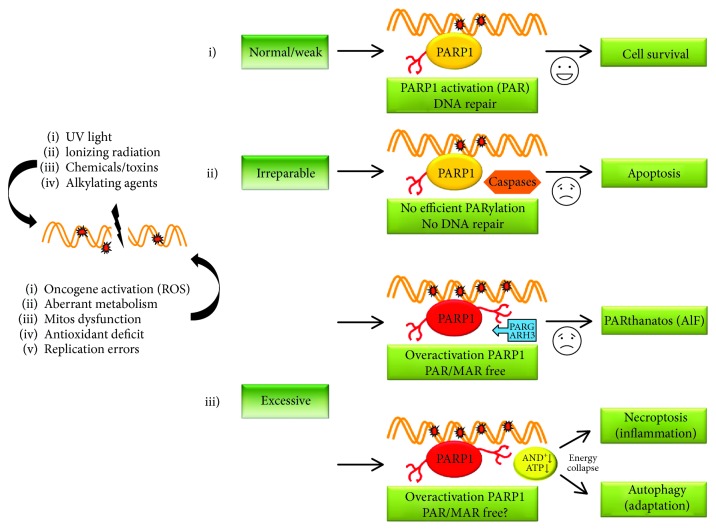
The intensity of the stimulus that induces DNA damage and the degree of activation of PARP1 determines the fate of a cell toward survival or death. *Destination 1*. When a slight damage occurs in DNA by endogenous or exogenous agents, PARP1 recognizes these damage points; PARylates itself, histones, and structural chromatin-related proteins; and finally promotes recruitment of DNA repair enzymes. If the DNA is repaired successfully, cells will survive. The effect on nuclear NAD^+^/ATP never leads to energy collapse. *Destination 2.* Multiple intense damage that despite the activity of PARP1 never will be repaired successfully. The cell enters in irreversible apoptosis, effector caspases 3 and 7 degrade PARP1 as the main competitor for the ATP, and the “eat-me” signal will appear on the cell surface. *Destination 3.* Excessive DNA damages, though not necessarily lethal, produce a phenomenon of overactivation of PARP1 and over-PARylation in nucleus. The energy consequences are lethal for cells, and the nuclear pool of NAD^+^/ATP will be seriously affected. Poly(ADP-ribose) glycohydrolases and ADP-ribose hydrolases mediate a rapid turnover and recycling of the bulk of PAR modification on nuclear PARylated proteins, autoPARylated PARPs, and free chains of ADP-ribose. The free monomers and little PAR chains are exported to cytosol to induce mitochondrial AIF translocation to nucleus. Finally, cell will die in a non-caspase-dependent process or PARthanatos. Overactivation of PARP1 consumes both NAD^+^ and ATP in nucleus triggering a high imbalance in the total pool of energy in the cells. This total energy collapse alters the functions of the main energy organelles such as mitochondria, ribosomes, or endosomes, favoring a total energy imbalance. At the same time, the presence of PAR chains and free ADP-ribose monomers in the cytosol modifies enzymes such as RIP1, triggering necroptosis. *As an alternative* to death by necroptosis, autophagy appears as an adaptive pathway to try to alleviate the energy crisis and prevent cell death arising. The nature of the stimulus and its durability will determine recycling of damage organelles, misfolded proteins, or lipid aggregates. PARylation could be considered a highly dynamic posttranslational modification of several proteins forming the autophagosome core or targeting intracellular components to be degraded. At the same time, cells are given an alternative source of energy until the damage is repaired or the stress situation ceases.

Attending the origin of DNA damages, the intensity and “durability” of these damages, and the activation levels of PARP1, we can define several destinations of the cells. When DNA damage is minimal, the recruitment of PARP1 to sites of DNA lesions activates the DNA damage response consuming a controlled amount of NAD^+^ and ATP. Depending on the type of lesion encountered, signaling mediated by molecules such as p53 and ATM or ATR promote cell cycle arrest, buying time for DNA repair enzymes to work [18, 19]. In this context, the energy expenditure and NAD^+^ required can be so great that the cells would enter almost immediately cell death or cellular “suicidal” pathways mediated by PARylation because even though the DNA damage is repaired, the collateral damage to the cellular energy machinery is too great (see Figure 1).

To link PARP1 (potent consumer of ATP and NAD^+^) energy collapse (oxidative phosphorylation to create ATP) and oxidative stress (collapse of mitochondrial ETC), several groups propose a theory in the form of a feedback loop: oxidative conditions lead to DNA damage, triggering PARP1 overactivation that promotes more energy collapse by over-PARylation and noncovalent MARylation that finally guide the cells to die. The suicidal over-PARylation disrupts the mitochondrial energy mechanisms, impairing the antioxidant capacity of the Krebs cycle and favoring the liberation of proapoptotic mitochondrial factors [20].

PAR-mediated cell death pathways can be subdivided into two subcategories, depending on the Poly(ADP-ribose) synthesis levels associated with the process (see Figure 1): (1) Low PAR synthesis cell death: PARP1 and “suicidal” proteases. Apoptosis is a nonreversible cell death pathway characterized by activation of caspases, membrane depolarization, exportation of mitochondrial death precursors, and nuclear disintegration. Apoptosis involves a biochemical expense which irreversibly leads to cell death. PARP1 is a preferred substrate for several “suicidal” proteases (caspases, calpains, cathepsins, granzymes, and matrix metalloproteinases (MMPs)). The proteolytic action of these proteases on PARP1 produces several specific cleavage fragments with different molecular weights. Each fragment could be associated with specific stress situations inside the cells or cell death programs [21]. When DNA damage is irreparable or the signaling by PARP1 does not lead to an optimal repair, cells enter irreversibly in cell death by apoptosis. In the initial steps of apoptosis, caspases 3 and 7 cleave the DNA-binding domain from the catalytic domain of PARP1 resulting in the inactivation of PARP1 and blocking the ATP competition between PARPs and caspases [21]. (2) Overactivation of PARP1 and cell death: PARthanatos and necroptosis. In response to intense and sustained damage, a large amount of Poly(ADP-ribose) is synthesized. The overactivation of PARP1 collapses the cellular energetic machinery and triggers necrosis, translated in proinflammatory conditions in the tissues. An indeterminate amount of Poly(ADP-ribose) could be exported to the cytosol and enter the mitochondria, promoting PARylation on AIF proteins (apoptosis-inducing factor). PARylated-AIF enzymes translocate from the mitochondrion to the nucleus, triggering chromatin condensation and DNA fragmentation into large fragments (~50 kb) which irreversibly leads to cell death called PARthanatos [22]. Actually, new studies have demonstrated in DNA double-strand break-dependent energy depletion models that the bioenergetic changes are adaptively regulated during PARthanatos, especially under macroautophagy deficiency [23].

For decades, necrosis has been considered a passive and unregulated process, linked in many cases to overactivation of PARP1 that leads the cell to a scenario of energy collapse, oxidative chaos, and alteration of biomembranes. Current studies have revealed several models of cell death with characteristics of necrosis but are certainly regulated by various proteins. A clear example is necroptosis mediated by RIP1 in response to TNF*α* death ligand released during inflammatory conditions. Upon binding to TNFR1, TRADD, TRAFs, RIP1, and cIAPs, proteins are recruited to form complex I blocking apoptosis and leading to expression of proinflammatory cytokines. In this model, the overactivation of PARP1 is considered a central element causing depletion of NAD^+^/ATP, releasing cathepsins and favoring AIF translocation. Furthermore, RIP1 protein has been proposed as a clear acceptor of PAR chains. However, there are many unanswered questions about whether necroptosis will be considered or not such as a programmed cell death mechanism [24, 25]. Autophagy is considered a housekeeping mechanism to recycle long-lived protein, aberrant organelles, and unfolded proteins and blocking intracellular pathogen invasion. Autophagy is generally thought of as a survival mechanism, although its deregulation has been linked to nonapoptotic cell death. The goal of necroptosis is to eliminate unnecessary or abnormal cells from the body under stress situations, metabolic diseases, or other pathological scenarios. Thus, a controlled recycling of damaged organelles in response to energy deletion under PARP overcativation palliate inflammatory response in tissues controlling the levels of necroptosis and PARthanatos [25]. The idea of autophagy as a mechanism for maintaining energy homeostasis under a context of activation of PARP proteins will be taken up in different sections of this review.

## 4. Autophagy in Mammalian Cells: Concept, Types, and Functions

Macroautophagy (referred to simply as “autophagy”) is an evolutionary ancient homeostatic “self-eating” pathway that has been highly conserved among eukaryotic cells. Autophagy is a catabolic lysosomal-associated process that targets intracellular components, from small portions of the cytosol, macromolecules, and unwanted organelles to chaperone-associated cargoes. Morphologically, this pathway is characterized by active membrane trafficking through formation of a unique double-membrane structure, called autophagosome, where engulfed cytosolic structures are delivered to the lysosome for bulk degradation [26, 27]. Autophagy plays a housekeeping role in the turnover of long-lived proteins, disposal of damaged organelles, and clearance of aggregate-prone proteins. The main objective is the adaptation and survival of the cell to the changing environment and finally to maintain the normal growth cellular fate [28]. Considering the origin of the subcellular material to be removed, we will refer to “basal” autophagy, which describes a nonstop removal of specific cargo, including unfolded or aggregated proteins, lipid droplets, or whole organelles. Thus, macroautophagy is the most selective autophagy pathway and is characterized by the formation of double-membrane intracellular phagosomes. “Stress-induced autophagy” normally is induced by nutrient deprivation or hypoxia and depends on the availability of autophagomes; however, deprivation of nutrients, oxygen, or growth factors does not exclude the activation of selective autophagy towards a specific cellular substrate with the ultimate goal of obtaining energy and basic components (amino acids, nucleosides, or lipids) used in cell maintenance and survival [29, 30].

Macroautophagy is interconnected with different pathways which regulate nutrient uptake, cell growth, and cell death programs. At the tissue level, autophagy should be considered an active player in physiological processes such as brain development, lineage differentiation, tissue architecture, and regulation of energy homeostasis of important organs (liver, brain, or heart).

It is widely demonstrated that extracellular proteins are degraded in the lysosomes by phagocytosis, pinocytosis, or endocytosis; however, intracellular unfolded or long-lived proteins could be degraded and recycled into amino acids by macroautophagy (proteins are engulfed in autophagosomes and degraded after fusion with lysosomes), microautophagy (soluble cytosolic proteins are directly internalized through the membrane of the lysosomes), and chaperone-mediated autophagy (CMA) (chaperone-dependent selection of proteins that are then targeted to lysosomes and directly translocated across the lysosome membrane for degradation) to maintain ATP levels compatible with cell survival. Different types of ordered organelle-selective autophagy pathways have been described and characterized as new macroautophagy examples, and in all of them, the active flux of biomembranes to engulf portions of cytosol is the main morphological characteristic. Many groups have described endothelial reticulum or ER-phagy, mitophagy (mitochondria), and ribophagy (ribosomes). Recent studies have determined selective autophagosome formation, engulfing lipid droplets (lipophagy) or toxic protein aggregates (aggrephagy) [28, 31].

The consequences of defects in autophagy for diseases are apparent, with growing evidence linking the mutation or loss of function of key autophagy genes to cancer, neuropathies, heart disease, autoimmune diseases, and inflammation. In cancer biology and tumor progression, it remains controversial when considering autophagy as beneficial or harmful for tumor cells and consequently for the growth and expansion of the tumor. Autophagy may act as a tumor-suppressive pathway, promoting cell cycle arrest or limiting necrosis and inflammation, or as a prooncogenic pathway, favoring cell survival in the presence of stressful conditions [29, 32]. The impact that autophagy causes in growth, development, and expansion of a tumor can be summarized as follows: tumor-initiating events (oncogene activation, impaired DNA repair pathways, or high metabolic defaults) promote cell proliferation, but also apoptosis, which limits tumor growth. Tumor growth is initially limited by the absence of a blood supply which can trigger autophagy-mediated survival in the most metabolically stressed tumor regions, commonly the hypoxic and starved center [33]. The eventual recruitment of a blood supply prevents hypoxia, provides glucose and amino acids, and reduces metabolic stress. In tumors enriched by cells with defects in both apoptosis and autophagy, necrotic cell death is stimulated in metabolically stressed tumor regions and this necrosis is associated with the activation of an inflammatory response, DNA damage, and tumor progression. Thus, autophagy will play a dual role, promoting survival, and adaptation to try to survive or in the opposite case could be used as a “Trojan Horse” of these cancer cells, inhibiting angiogenesis, promoting more selective apoptosis, and favoring the “eat-me” signals [33].

In other diseases such as neuropathies (Huntington's, Alzheimer's, and Parkinson's diseases) and ischemic heart disease, autophagy is more widely accepted as beneficial given its role in eliminating toxins, aberrant structures, and promoting cell viability. Most neurodegenerative disorders are characterized by the accumulation of misfolded proteins that coalesce into “inclusions” and become visible under the light microscope in the brains and spinal cords of affected patients. The high sensitivity of mature neurons to misfolded protein stress is well known, impairing the right neuronal functions, promoting neuron cell death and neurodegenerative disorders. *In vivo* animal models of neuropathies have demonstrated that basal levels of autophagy are required for the continued health and normal function of neurons [34, 35]. Autophagy plays a key role in cardiomyocyte growth and satisfactory heart activity. Autophagy may antagonize ventricular hypertrophy by increasing protein degradation, which decreases tissue mass in ischemic mouse models. However, the rate of protective autophagy declines with age, demonstrating an eventual autophagy/aging crosstalk.

## 5. Molecular Machinery of Autophagosome Formation

**Figure 2:**
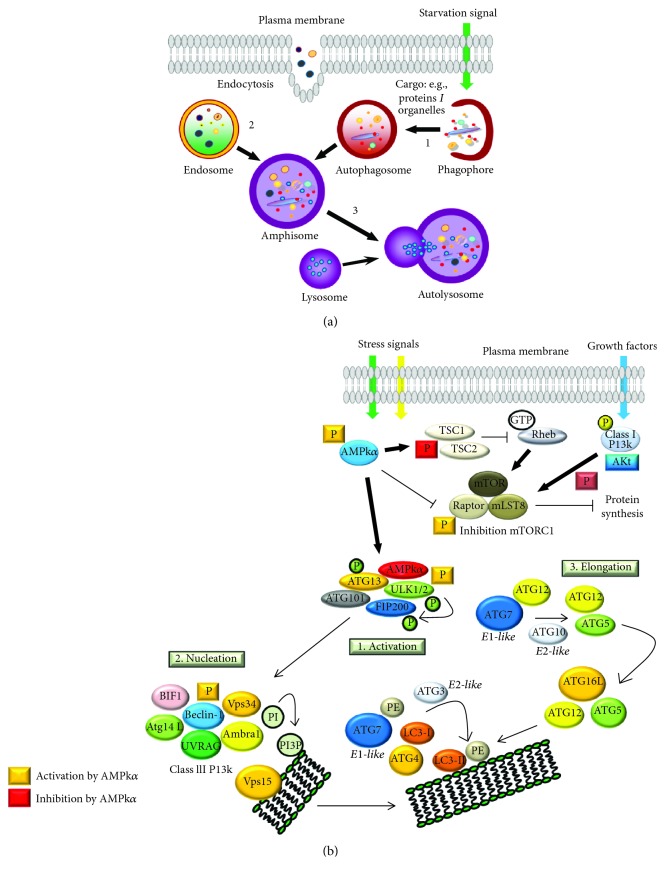
Autophagy steps. (a) Membrane traffic associated to autophagosome formation. During autophagy, sequestration begins with the formation of a phagophore that expands into a double-membrane autophagosome. (1) Stages of nucleation and elongation: the autophagic charge will be engulfed by a double-lipid membrane called phagophore; the phagophore suffers elongation and closure to form an autophagosome. (2) This autophagosome may, or not, fuse with an endosome to form an amphisome. (3) Finally, the amphisome will fuse with lysosomes to form an autolysosome; in this step, acid hydrolases degrade the content of the autolysosome. Finally, the content may be recycled through permeases that efflux the content to the cytosol. (b) Vesicle induction and phagosome creation. In response to stress signals (starvation, hypoxia, and growth factor depletion), AMPk is activated and mTORC1 is inhibited, leading the stimulation of the ULK1 core (activation). ULK1 kinase will phosphorylate Beclin-1 leading to VPS34 activation and the initiation of phagophore formation (nucleation). ATG5-ATG12 conjugation involves ATG7 and ATG10 promoting an “E-like ligase” reaction of ATG12-ATG5-ATG16L influencing on the phospholipidic elongation of the double membranes. The complex ATG12-ATG5-ATG16L acts like an E3-function towards the LC3-PE assembly (LC3-II isoform) (elongation). Autophagosome maturations also involve fusion with lysosomes, degradation and recycling of nutrients and metabolites, and recycling of LC3-I isoform. This membrane trafficking maintains the same molecular events in nonselective and organelle-specific autophagy (mitophagy, ribophagy, and selective formation of amphisomes).

In mammalian cells, autophagy sequestration features important membrane traffic and begins with the active formation of omegasome structures on the rough endoplasmic reticulum called phagophores. Phagophores expand into double-membrane autophagosomes while surrounding a portion of the cytoplasm. Autophagosomes may fuse with endosomes (product of endocytosis from the external microenvironment) considering itself heterophagy (the cell is able to internalize and degrade the material that originates from outside of the cell). The newborn organelles are called amphisomes. The final destination of autophagosomes or amphisomes is to fuse with several lysosomes, which supply acid hydrolases. In this new cellular compartment or autolysosome, the engulfed cargo will be recycled into macromolecules which are released in the cytosol (see Figure 2(a)).

One of the elusive fields of autophagy is deciphering the molecular details of autophagosome biogenesis. In many cases, the sequestration step is the most complex part of autophagy because the cytoplasm must be segregated, often in a direct or specific manner, and moved from the intracellular space into the vacuole or lysosome lumen. Initiation of double-membrane phagophores requires a specific cascade of proteins, kinases, and E-like ligases to allow the recruitment, formation, and sequestration of intracellular portions.

The journey into the molecular realm of autophagy began with the identification of a set of evolutionary conserved genes termed autophagy- (ATG-) related genes. Independent genetic screens in yeast model systems have identified 38 ATG genes which are involved in various subtypes of macroautophagy, including starvation-induced autophagy, the cytoplasm-to-vacuole targeting (Cvt) pathway, and pexophagy. Many of the genes have known orthologues in other eukaryotes [36, 37].

In yeast, approximately 20 Atg proteins are considered essential in the initial steps of phagophore formation and closuring of autophagosomes. These proteins have been classified into five functional groups based on identified protein-protein interaction: Atg1 kinase complex, autophagy-specific class III phosphatidylinositol 3-kinase or PI(3)K complex, Atg9-Atg2/Atg18 complex, Atg12 conjugation complex, and Atg8 conjugation system. Extensive research has demonstrated functional interactions between them in the core of newborn autophagosomes [38]. In mammalian cells, the number is higher, including an intricate number of 400 members from different families. Classically, an integrated view of mammalian autophagy establishes several steps in the final double-membrane vesicle formation and engulfment of the cargo [39, 40]. In this review, we focus on the phagophore formation step, briefly describing the discovery and functions of the key players sensing stress situations and promoting the biochemical organization of double-membrane engulfing proteins, organelles, or portions of the cytosol. The implication of the PARylation process in the regulation of the initial stages of autophagy is analyzed in detail below.

## 6. Vesicle Induction: Phagophore Formation—Regulation by AMPk and mTORC1

Autophagy disassembles unnecessary or dysfunctional components, resulting in a highly modulated catabolic pathway in response to several physiological cell stresses and pathological situations. The delicate balance between external energy and nutrient supply and internal production and consumption is a demanding task in the cells. There are two interconnected proteins with the autophagy core-regulating signaling network: AMPk and mTORC1. Both proteins have the capacity to sense ATP and nutrient availability, modulating the activity of the main ATG1 functional orthologue in autophagosome closuring, called ULK1 (unc-51-like autophagy-activating kinase 1), in mammals.

AMP-activated protein kinase (AMPk) is a highly conserved kinase considered to be the major energy sensor in eukaryotic cells (sensing increases in intracellular AMP/ATP and ADP/ATP ratios). AMPk is a serine/threonine kinase that negatively regulates several enzymes of the lipid metabolism and activates different catabolic processes in eukaryotic cells such as glucose uptake and metabolism, increasing ATP generation pathways and decreasing ATP consumption pathways. The balance of ATP synthesis/consumption allows the maintenance of energy homeostasis, makes the energy distribution into growth fates adequate, and triggers several adaptive cellular programs during stressful situations. Structurally, AMPk is a trimeric protein which presents three differentiated domains: a catalytic subunit (*α*) and two regulatory subunits (*β* and *γ*). Different groups have determined the crystal structure of several holoenzymes of AMPk [41, 42]. The *α* subunit is encoded by two isoforms, while the regulatory subunits present three isoforms in which expression and combination into the AMPk structure are dependent on the cell type. The most widely expressed isoforms of AMPk are AMPk*α*1, AMPk*β*1, and AMPk*γ*1; however, other isoforms are more restricted to specific tissues [42]. Under nutrient deprivation, hypoxia, DNA damage, or mitochondrial failure, the AMP/ADP : ATP ratio is sensed by LKB1 (liver kinase B1) which promotes the regulatory phosphorylation of Thr172 on the AMPk*α* subunit. Under this context, AMPk triggers catabolic processes in order to restore the ATP levels through the breakdown of different macromolecules [41, 42].

The mammalian target of Rapamycin (mTOR) is the major nutrient sensor and a central regulator of growth and metabolism in the cell, involved with processes including angiogenesis, autophagy, and protein metabolism. mTOR is a ubiquitously conserved serine/threonine kinase which represents the molecular core component of two multisubunit complexes, mTORC1 and mTORC2. mTORC1 senses the availability of amino acids, oxygen, and growth factors in basal and stressed conditions, promoting cell growth and anabolic pathways. On the other hand, mTORC2 is considered mainly as a regulator of the organization and rearrangement of the cytoskeleton [43]. Structural differences between mTORC1 and mTORC2 determine the sensitivity to Rapamycin. mTORC1 basically is formed by three subunits, mTOR kinase (the functional unit), RAPTOR (a regulatory protein sensitive to inhibition by Rapamycin), and mLST8 (mammalian lethal with Sec13 protein 8). The biochemical regulation of the mTORC1 activity is highly dependent on the tumor suppressor TSC2, an upstream component of the mTORC1 complex. TSC2 contains a GTPase domain that inactivates the small Ras-like GTPase Rheb, which has been shown to associate and activate the mTORC1 complex. Loss of TSC2 (and TSC1) leads to overactivation of mTORC1, triggering translation (by p70^S6k^ and 4E-BP1 regulation), cell cycle, and cell growth (see Figure 2(b)). mTORC2 exhibits the mTOR kinase and mLTS8 subunits and also DEPTOR (DEP domain-containing mTOR-interacting protein), and in this complex, RAPTOR is replaced by RICTOR (Rapamycin insensitive companion of mTOR) which is not sensitive to Rapamycin. Under energy and growth factor availability, mTORC2 controls cellular metabolism and cytoskeleton dynamics (by Akt/GSk3*β* activation).

In terms of autophagy regulation, AMPk is considered the major positive regulator of autophagy (catabolism) while mTORC1 is considered the major negative regulator of phagophore induction (anabolism). Under a proautophagic scenario in the cells such as amino acid starvation or energy depletion, binding of AMP or ADP to the *γ* subunit of AMPk promotes Thr172 phosphorylation by LKB1 in the *α* subunit and full activation of AMPk kinase. Recent studies suggest that AMPk may also be a redox-sensing protein. Reactive oxygen species (ROS) are naturally produced by many metabolic reactions, notably by the production of ATP in the mitochondria, and the strict control of their levels is important for cellular homeostasis. ROS can indirectly activate AMPk through increases in AMP or by post translational modifications of the *α* subunit of AMPk [44]. AMPk potently promotes autophagy inhibiting mTORC1 through phosphorylation of TSC2 and indirectly blocks GTPase Rheb-positive regulation on mTOC1 activity. In this context, protein synthesis and several anabolic pathways are inhibited; in a second step, AMPk binds the inactive autophagy core ULK1 complex (formed by ULK1 kinase, ATG13 regulator, ATG101/FIP200 which is a key component of the autophagy initiation process) and it phosphorylates the component ULK1/2 kinase. As a consequence of a cascade of autophosphorylations between ULK1/2 and ATG101/FIP200, ULK1 stimulates autophagy initiation.

The ULK1/2 complex translocates to the phagophore localization, where it activates the class III phosphatidylinositol 3-kinase (class III PI3k) complex composed of VPS34 (vacuolar protein sorting 34 or PI3k enzyme), Beclin-1, VPS15, and ATG14 proteins. The kinase activity of ULK1/2 enhances the VPS34 activity by direct phosphorylation on Beclin-1. Class III PI3k activity is inhibited when Beclin-1 is bound to Bcl-2 [45] but is stimulated upon UVRAG recruitment to the complex [46]. Interestingly, Ambra1 also directly binds Beclin-1 to regulate the stability of Beclin-1/PI3k complex formation, competing under specific proautophagic condition with Ambra1 to stabilize the complex [46]. These events lead to autophagosome formation following the extension and closure of the mature autophagosomes (see Figure 2(b)). AMPk presumably suppresses nonessential vesicle trafficking in favor of membrane trafficking into the autophagy pathway during nutrient starvation in a VPS15/VPS34 class III phosphatidylinositol 3-kinase or PI (3) kinase-dependent manner [42, 47].

Following the nucleation step, other Atg proteins are recruited to the membrane of the preautophagosomes to promote the elongation and expansion of these newborn organelles. Elongation membranes require Atg3, Atg4, Atg7, Atg10, and an Atg5-Atg12-Atg16L complex to conjugate phosphatidylethanolamine (PE) to the microtubule-associated protein 1 light chain 3- (LC3-) I to form LC3-II [48]. The lipidated isoform LC3-II translocates from cytoplasm to the membrane of the preautophagosomes. Once the autophagosomes are closed, engulfing organelles, proteins, lipid droplets, or pathogens, a large majority of Atg proteins are released from the surface of autophagosomes to begin a new round of nucleation and membrane elongation. Currently, there is controversy about the amount of isoform LC3-II that is released and recycled from mature autophagosomes, given that a percentage of LC3-II is exposed on the inner side of the membranes and therefore would be subject to the action of lysosomal acid hydrolases [49].

## 7. DNA Damage Response and Autophagy: A Survival Association

In response to DNA damage induced by ROS (or reactive nitrogen species (RNS)), cells activate a high number of pathways in order to repair and maintain genome integrity and mediate survival pathways. Several kinds of proteins are implicated in DNA damage responses (DDRs) which are considered sensors (recognize and signal the damage), mediators, and effectors (repair the damage). Mediators and effectors are molecules that transduce nuclear signals to the cytosol where several processes are activated in order to better face adverse conditions. During DDRs, two events must be controlled in order to promote adaptation and survival: cell cycle checkpoints are activated to block proliferation and allow repair, and cell death pathways must be “kept on alert” in case of any unrepaired or excessive DNA damage.

ROS have been repeatedly reported as early inducers of autophagy due to their ability to produce oxidation of proteins, alteration of biological membranes, and DNA damage. The DNA lesions induced by ROS are involved in mutations, cancer, and many other diseases. PARPs are pivotal guardians in maintaining the integrity of the genome and triggering diverse kinds of metabolic strategies to escape from these adverse conditions. Currently, there are several groups that have demonstrated an active connection between the events that lead to DNA repair and the induction of autophagy. Accumulating evidence suggests that autophagy can be activated by DNA damage. ATM, a DNA damage-activated kinase, has been described as an important link between the DNA damage response (DDR) and the induction of autophagy. ATM binds to double-strand breaks (DSBs) in conjunction with the MRN complex and undergoes autophosphorylation and activation. In turn, ATM activates various downstream effector proteins, including Chk2 and Chk1 involved in cell cycle control or the tumor suppressor p53 which regulates cell survival versus death and HDAC1 and HDAC2 which are responsible for chromatin remodeling [50]. In response to DNA damage by mitochondrial ROS, external toxins, or irradiation, ATM is autophosphorylated within a MRN multiprotein complex that binds DSBs. Activated ATM initiates a pathway that results in activation of AMPk and its target TSC2 which functions as an inhibitor of mTORC1, promoting ULK1-dependent autophagosome formation. In addition, ATM directly phosphorylates and stabilizes p53 which transcriptionally regulates various regulators of the autophagic pathway including AMPk (energy sensor and autophagy activator) in colon cancer cells and during spermatogenesis, DAPK1 (death-associated protein kinase 1 or regulator of cell death and autophagy), and PTEN (phosphatase and tumor suppressor) in hepatocarcinoma cells and other cancer cell lines [51–53].

Recent studies have shown that treatment of certain types of tumor cells with genotoxic agents activate AMPk in a nucleus-independent way. Etoposide activates specifically the isoform AMPk*α*1 and not the *α*2 isoform, primarily within nucleus. AMPk*α*1 activation is independent of ATM signaling during etoposide-dependent DDRs. In this model, etoposide increased the intracellular Ca^+2^ levels and leads the activation of CaMkk2 kinase. AMPk*α*1 protected tumor cells against etoposide by limiting entry into the S-phase [54]. Considering the high energy cost and NAD^+^ involved during nuclear overactivation of PARP1 in response to DNA damage, regardless of whether the stimulus has a proautophagic character or not, we must explain that in most DDR models and autophagy described in normal and tumor cells, the activation of PARP1 has triggered a highly regulated process of autophagy aimed at survival. Therefore, it is obvious to think that treatment combined with PARP inhibitors inside a context of compromised autophagy could be considered as an interesting and novel antitumor therapy.

Several studies propose a role of PARP1 in the regulation of autophagy in response to DNA damage. Different models of MEF 3T3 *KO* for PARP1 and in combination with PARP inhibitors (PARPi) (PJ34, DPQ, or Olaparib) were used by different groups to describe controlled overactivation of PARP1 and highly modulated prosurvival autophagy in response to alkylating and intercalating agents or ionizing radiation. Doxorubicin treatment leads to overactivation of PARP1, followed by ATP and NAD^+^ depletion that trigger the nontoxic accumulation of autophagosomes in a model of MEF *parp1*^+/+^ cells. An effective *KO* model for PARP1 (MEF *parp1*^−/−^) or the treatment of MEF *parp1*^+/+^ with PARPi increased the sensitivity of the cells to Doxorubicin, promoting high and uncontrolled levels of cell death. On the other hand, pharmacological or genetic inhibition of autophagy in a PARP1 KO model resulted in increased necrosis, suggesting a PARP1-mediated protective role of autophagy in response to chemotherapy [55]. A study in *Bax*^−/−^*Bak*^−/−^ double knockout MEFs has elucidated the signaling pathway and biological function of autophagy induced by MNNG, a commonly used DNA-alkylating agent. In response to MNNG, double *KO* MEFs activated PARP1, reducing intracellular ATP levels and triggering the AMPk pathway and mTORC1 suppression. As a result, there was an accumulation of autophagosomes and cells showed Poly(ADP-ribose) profiles and persistent resistance for a long time of treatment. Suppression of the AMPk pathway blocked MNNG-induced autophagy and enhanced cell death [56]. Finally, other studies obtained the same conclusions in a nasopharyngeal carcinoma model exploring overactivation of PARP1, PARylation-dependent energy depletion and upregulation of the AMPk and ULK1 pathways in CNE-2 carcinoma cells upon ionizing radiation [57].

Many genotoxic agents activate AMPk kinase. Depending on the type of agent that induces DNA damage, the downstream cell response mediated by AMPk will be different. In tumor biology, the DNA-dependent AMPk activation could be considered protumorigenic promoting cell viability/survival or antitumorigenic cells would be more vulnerable to genotoxic stress. All these studies have demonstrated that autophagy should be considered as an important target in cancer during the induction of DNA damage, and consequently, new strategies based on the concept of synthetic lethality of PARPi must be explored.

## 8. ROS-Induced DNA Damage Leads to PARP1-Mediated AMPk Activation during Starvation-Induced Autophagy

Cancer cells require a continuous source of nutrients and oxygen, which is supplied through the growth of new blood vessels, providing the tumor with nutrients and evacuating metabolic wastes. At the cellular level, the exchange of nutrients, oxygen, and growth factors with the intracellular medium is a crucial process given that the absence of nutrients (starvation) or the deficiency of oxygen (hypoxia) can induce metabolic stress, oxidation of biomolecules, DNA damage, and PARP1 activation, situations in which cells activate autophagy as an adaptation and survival pathway. In this context, one initial signal during starvation-induced autophagy involves the activation PARP1 and the posttranslational modification by Poly(ADP-ribosyl)ation of several autophagy machinery proteins, favoring the autophagosome closure.

A large amount of evidence has demonstrated that starvation-induced autophagy is delayed in the absence of PARP1 in different models of normal and tumoral cells. Chemical inhibitors of autophagy as 3-methyl adenine (3-MA) (inhibitor of class III PI3k) or siRNA-based knockdown of ATG7 (nucleation of autophagosomes) completely prevented autophagy in *parp1*^−/−^ 3T3 MEFs under starvation conditions. These data demonstrated that the absence of PARP1 synergizes with 3-MA or genetic knockdown to suppress autophagy during starvation. On the other hand, chemical inhibition of Poly(ADP-ribosyl)ation with several PARPi or specific silencing by siRNA of PARP1 reduced the percentage of *parp1*^+/+^ 3T3 MEFs showing impaired membrane trafficking and LC3 translocation during autophagosome formation. The importance of controlled Poly(ADP-ribose) accumulation, due to PARP1 overactivation, in the initial steps of starvation-induced autophagy has been demonstrated [58]. Concomitant elimination of Poly(ADP-ribose) glycohydrolase (PARG) (enzyme degrading Poly(ADP-ribose) and ATG proteins1 (as ATG7, ULK1, or ATG5) has demonstrated that PAR accumulation after nutrient deprivation does not compromise cell viability; thus, the increased levels of autophagy are not ascribed to a cellular attempt to detoxify the excess of the PAR polymer in autophagosomes. There may be a mechanism of fine-tuning in the induction of PARylation-mediated autophagy [58, 59].

**Figure 3:**
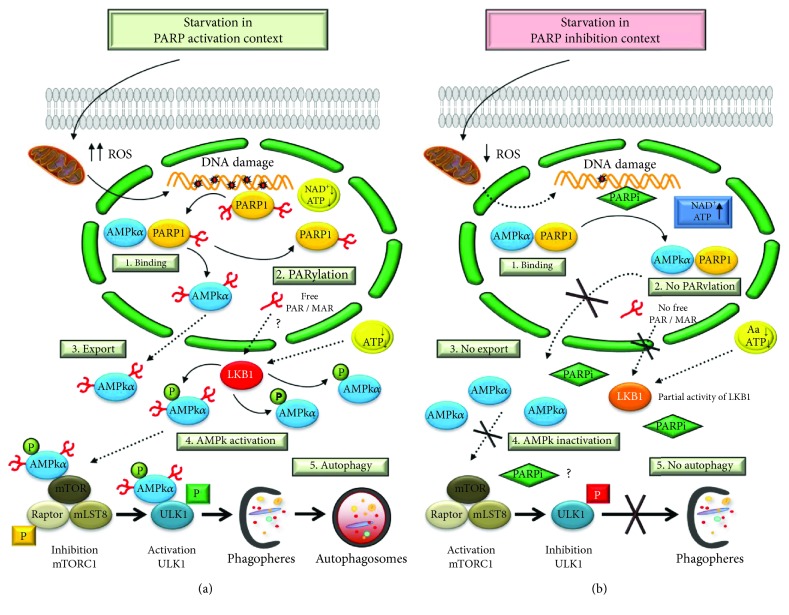
(a) PARylation regulates autophagy through AMPK*α* activation. PARP1 forms a complex with AMPK*α* in nucleus (1). During the starvation-induced autophagy, ROS production induces DNA damage and overactivation of PARP1. Auto-PARylated PARP1 is able to modify by PARylation in the AMPK*α*1 subunit (2). The complex is disrupted and PAR-AMPK*α* is exported to cytosol (3). The presence of PAR-AMPK and the continuous absence of amino acids and ATP depletion favor total activation of AMPK*α* population by LKB1, inhibition of mTORC1, interaction PAR-phospho-AMPK/ULK1, and autophagosome formation (4). LKB1 activity is presumably modified in a PARylation-dependent manner. (b) Starvation-induced ROS production was abrogated during the treatment with PARP inhibitors. Following AMPK*α*1/PARP1 interaction (1), the AMPK*α*1 subunit is not PARylated and the nuclear export of AMPK is inhibited (2 and 3). In spite of nutrient and energy depletion, AMPK*α* is inhibited; mTORC1 is partially activated and interacts with ULK1 favoring its inhibition (4). Finally, the autophagosomes production will be delayed.

The origin of PARP1 activation during starvation focuses on the mitochondrial production and nuclear translocation of ROS. Under these oxidative conditions, DNA damage is recognized by PARP1, leading to PAR synthesis and triggering the initiation of autophagy. Although PARP1 knockout cells also produce ROS during starvation, this production does not lead to massive DNA damage and PARP1 activation. Consequently, these cells display an impaired starvation-induced autophagy [58] (see Figure 3(a)).

How is PARP1 able to modulate the cytosolic assembly of autophagosomes? Most classical studies have characterized DNA-dependent PARPs as genome integrity maintenance enzymes enclosing their activity in the nucleus and highly fixed in DNA functions. However, different groups are demonstrating that the influence of PARP activity goes beyond the nucleus and directly impacts the main cellular metabolic pathways, anabolism and catabolism. In general, PARPs modulate NAD^+^ metabolism, energy pathways, and oxidative metabolism so we could conclude that inside a scenario of energy depletion guided by oxidative stress, PARPs potentiate cell scape or cell death pathways. Reversible PARylation is a pleiotropic regulator of various cellular functions but uncontrolled PARP activation may also lead to cell death. Moreover, noncovalent PARylation or MARylation could be considered as an effective cytosolic posttranslational modification of diverse MAPk kinases and mitochondrial cell death factors. To link PARP1 (potent consumer of ATP and NAD^+^) energy collapse (oxidative phosphorylation to create ATP) and oxidative stress (collapse of mitochondrial ETC), several groups propose a theory in the form of a feedback loop: oxidative conditions lead to DNA damage and trigger PARP1 overactivation that intensifies energy collapse by over-PARylation that finally guides the cells to die. The suicidal over-PARylation disrupts mitochondrial energy mechanisms, impairing the antioxidant capacity of the Krebs cycle and favoring the liberation of proapoptotic mitochondrial factors [20]. Autophagy must be considered a scape pathway restoring ATP levels and blocking or delaying cell death.

DNA damage derived from starvation-induced ROS triggers an important depletion in intracellular ATP levels by overactivation of PARP1. The role of PARP1 in starvation-induced autophagy is related to its ability to sense DNA damage and deplete energy stores after its overactivation. This energy collapse is sensed by LKB1, promoting AMPk activation and mTORC1 inhibition. Knockout cells or cells with inefficient PARP1 activity show a potent downregulation on specific phospho-Thr172 AMPk*α* by LKB1. In consequence, the mTORC1 targets p70^S6k^ and 4E-BP1 maintain their phosphorylation inhibiting autophagosome formation [58]. However, in this model, it is not possible to exclude the possibility of a perturbation in Ca^2+^/calcium-/calmodulin-dependent kinase kinase 2 (CaMKK2) flux after PARP1 ablation upstream of the mitochondria leading to altered ATP synthesis and AMPk activation [60].

A nuclear population of the *α* isoform of AMPk has been described to interact with PARP1 [35]. The modification of AMPk*α* by Poly(ADP-ribose) and the mutual interaction between PARP1 and AMPk*α* function as a multifaceted molecular switch to optimize the initiation of autophagy. In Figure 3, we summarize two demonstrated scenarios: (I) starvation in a PARP1 activation context: a well-defined, non-PARylated AMPk*α* population has been described in the nucleus in functional interaction with PARP1 in nonstarved cells. During nutrient deprivation, ROS exported from mitochondria induce DNA damage and PARP1 recognizes this damage; in order to promote DNA repair, PARP1 is over activated, consuming ATP and NAD^+^ as substrates to synthetize Poly(ADP-ribose). The energy depletion could be sensed by LKB1 kinases to initiate autophagosome formation. PARylation of AMPk*α* at the AMPk*α*/PARP1 complex is a key event in initiating autophagy. AMPk*α* is transiently PARylated, disrupting the complex with PARP1 and being exported from the nucleus to the cytosol. PARylated cytosolic AMPk*α* triggers the total activation by LKB1 of the cytosolic AMPk*α* pool. Finally, the total pool of active AMPk*α* inhibits the mTORC1 complex and activates the autophagy core ULK1 complex, favoring the nucleation and elongation of phagophores around cytosol portions in order to be degraded and recycled into amino acids and other essential biomolecules (see Figure 3(a)).

The specific Poly(ADP-ribosyl)ation of the *α* subunit is needed to undergo AMPk*α* nuclear export. Abolishing the active nuclear translocation of PARylated AMPk*α* or the genetically engineered mutation on putative PARyaltion sites blocks efficiently the nuclear export compromising the autophagosomes formation [59]. The interaction between PARP1 and AMPk*α* has been described for DNA damage-dependent PARP1 activation while PARP1 has been reported to be a target of AMPk*α* [61]. During proinflammatory situations, PARP1 is able to modulate the expression of Bcl-6 through its binding at Bcl-6 intron 1. Phosphorylation of PARP1 at serine 177 (Ser-177) by AMPk kinase promotes dissociation from Bcl-6 intron 1, increases Bcl-6 expression, and inhibits expression of inflammatory mediators, demonstrating an anti-inflammatory crosstalk linking AMPk and PARP1 activity [61].

To evaluate the possibility of a mutual interaction between AMPk*α* and PARP1 leading to PARP1 phosphorylation, starved cells treated with the AMPk kinase inhibitor compound C showed decreased PARylation of AMPk*α*, suggesting that full AMPk activity was needed for PARP1 activation during nutrient deprivation [59]. (II) Starvation in a PARP1 inhibition context: PARP1-deficient cells display a reduced production of ROS, even at very early time points following starvation [58]. This finding is consistent with previous results showing reduced ROS production in lymphocytes challenged with exogenous oxidative stress and treated with PARP inhibitors [62]. PARP1 knockout cells or cells treated with a PARP inhibitor show sharply reduced DNA damage levels, and the machinery to repair DNA damage is not as efficient as in PARP1 wild-type cells, resulting in a residual level of damage after long times of starvation of which the final consequence is compromised autophagy and prominent apoptosis cell death [58].

In PARP1-inactivated cells, the absence of efficient PARylation retains the stability of the PARP1/AMPk*α* complex and AMPk*α* is not transported from the nucleus to the cytosol during starvation. The final consequence is an inefficient activation of the cytosolic AMPk*α* pool, partially maintaining the activity of mTORC1 and seriously compromising the activation of ULK1 and the initiation of phagophores [59] (see Figure 3(b)).

The crucial role of neonatal autophagy was clearly demonstrated by targeted inactivation of the autophagy-related genes ATG5 and ATG7. Mice deficient in ATG5/ATG7 were apparently normal in birth, except for a slightly lower body weight than control or wild-type (approximately 10% in ATG5-null and 18% in ATG7-null mice). Moreover, ATG7-null animals presented deficiency in the liver causing hepatomegaly and hepatic cell swelling. The curve of survival of neonates demontrated that the null animals showed serious difficulties to growth and survive, indicating the important role of the proteins that regulate the formation of autophagosomes during embryonic development [63]. It is described that phenotypically, PARP1 knockout mice had the average litter size smaller than wild-type mice (approximately 20%). Several *in vivo* observations in starved neonates of PARP1 mutant mice showed decreased frequency of hepatic multimembrane lipid droplets, presumably autophagosomes engulfing mitochondria and cytosol portions, implying a physiological role of PARP1 in starvation-induced autophagy [58].

In conclusion, the nucleus and the functional interaction between PARP1 and AMPk*α* are initial and essential sensors of the metabolic alterations derived from perturbations in the nutritional extracellular status, not necessarily related with the alterations in genomic integrity.

## 9. Concluding Remarks

One of the most remarkable findings in our study is the need for the AMPk*α* nuclear export to perform its cytosolic function during the initial steps of starvation-induced autophagy, but how PARylated AMPk*α* coming out of the nucleus “hits” cytosolic AMPk*α* remains to be clarified. Nuclear sensors, including PARP1, detect perturbations in nuclear and genetic homeostasis to activate a mechanism to repair and, in case of failure, promote different types of cell death for the benefit of the organism homeostasis. Considering different studies, we could conclude that DNA lesions are potential activators of nonselective autophagy mechanisms. In this way, we have enough evidence to affirm that PARP1 and PARylation play a key role in autophagy, beyond the nuclear activation of PARP1.

This model has been demonstrated in normal and cancer cells, so efficient treatment with drugs on cancer cells opens a new, interesting, and novel field with PARP inhibitors and molecules targeting prosurvival pathways under physiological stimuli such as starvation, hypoxia, growth factor deprivation, etcetera.

## Figures and Tables

**Table 1:** Nomenclature, enzymatic activity, and biological functions of PARP proteins.

PARP	Alternative names	Demonstrated activity	Cellular localization	Biological processes
PARP1	ARTD1	PARylation	(i) Nucleus	(i) DNA repair (ii) Genome integrity (iii) Transcription (iv) Replication (v) Cell cycle (vi) Metabolism and development (vii) Proteasome degradation (viii) Diseases: cancer, inflammation, and HIV

PARP2	ARTD2	PARylation	(i) Nucleus	(i) DNA repair (ii) Genome integrity (iii) Transcription (iv) Cell cycle (v) Metabolism (vi) Cancer/inflammation

PARP3	ARTD3	MARylation	(i) Nucleus	(i) DNA repair (ii) Genome integrity (iii) Cell cycle (iv) Development (v) Cancer

PARP4	ARTD4/VPARP	MARylation	(i) Nucleus (ii) Exosomes (iii) Cell membrane	(i) Vault biology (ii) Cancer

TNK1	ARTD5/PARP5A	PARylation	(i) Nucleus (ii) Telomeres (iii) Golgi (iv) Cytoplasm	(i) Mitotic spindle (ii) Telomere maintenance (iii) Metabolism

TNK2	ARTD6/PARP5B	PARylation	(i) Nucleus (ii) Telomeres (iii) Golgi (iv) Cytoplasm	(i) Inflammation (ii) Telomere maintenance? (iii) Metabolism

PARP6	ARTD17	Not determined	(i) Cytoplasm?	(i) Cell proliferation

PARP7	ARTD14/TIPARP	MARylation	(i) Nucleus? (ii) Cytoplasm?	(i) Transcription (ii) Antiviral effects (iii) Cytosolic RNA processing

PARP8	ARTD16	Not determined	(i) Not determined	(i) Unknown

PARP9	ARTD9/BAL1	Inactive/unknown	(i) Nucleus (ii) Cell membrane (iii) Cytoplasm?	(i) Cell migration (ii) Tumor formation

PARP10	ARTD10	MARylation	(i) Nucleus (ii) Cytoplasm	(i) Cell proliferation (ii) Transcription (iii) Cytosolic RNA processing

PARP11	ARTD11	Not determined	(i) Unknown	(i) Unknown

PARP12	ARTD12	MARylation	(i) Cytoplasm	(i) RNA processing

PARP13	ARTD13/ZAP/ZC3HAV1	Inactive/unknown	(i) Cytoplasm	(i) RNA processing

PARP14	ARTD8/BAL2	MARylation	(i) Nucleus (ii) Cell membrane	(i) Inflammation (ii) Transcription (iii) Metabolism (iv) Tumor formation (v) Nuclear RNA processing

PARP15	ARTD7/BAL3	MARylation	(i) Cytoplasm	(i) Cytosolic RNA processing (ii) Tumor formation

PARP16	ARTD15	MARylation	(i) Cell membrane (ii) ER	(i) Unfolded protein response (UPR)
